# Endoscopic repair of cerebrospinal fluid rhinorrhea^☆^

**DOI:** 10.1016/j.bjorl.2016.04.024

**Published:** 2016-06-04

**Authors:** Vladimir Kljajić, Petar Vuleković, Ljiljana Vlaški, Slobodan Savović, Danijela Dragičević, Vladimir Papić

**Affiliations:** a University of Novi Sad, Faculty of Medicine, Hajduk Veljkova, Novi Sad, Serbia; b Clinical Center of Vojvodina, ENT Clinic, Hajduk Veljkova, Novi Sad, Serbia; c Clinical Center of Vojvodina, Clinic of Neurosurgery, Hajduk Veljkova, Novi Sad, Serbia

**Keywords:** Cerebrospinal fluid rhinorrhea, Nasal surgical procedures, Endoscopy, Fistula, Fluorescein, Treatment outcome, Rinorreia de líquido cerebrospinal, Procedimentos cirúrgicos nasais, Endoscopia, Fístula, Fluoresceína, Desfecho do tratamento

## Abstract

**Introduction:**

Nasal liquorrhea indicates a cerebrospinal fluid fistula, an open communication between the intracranial cerebrospinal fluid and the nasal cavity. It can be traumatic and spontaneous.

**Objective:**

The aim of this study was to assess the outcome of endoscopic repair of cerebrospinal fluid fistula using fluorescein.

**Methods:**

This retrospective study included 30 patients of both sexes, with a mean age of 48.7 years, treated in the period from 2007 to 2015. All patients underwent lumbar administration of 5% sodium fluorescein solution preoperatively. Fistula was closed using three-layer graft and fibrin glue.

**Results:**

Cerebrospinal fluid fistulas were commonly located in the ethmoid (37%) and sphenoid sinus (33%). Most patients presented with traumatic cerebrospinal fluid fistulas (2/3 of patients). The reported success rate for the first repair attempt was 97%. Complications occurred in three patients: one patient presented with acute hydrocephalus, one with reversible encephalopathy syndrome on the fifth postoperative day with bilateral loss of vision, and one patient was diagnosed with hydrocephalus two years after the repair of cerebrospinal fluid fistula.

**Conclusion:**

Endoscopic diagnosis and repair of cerebrospinal fluid fistulas using fluorescein intrathecally has high success rate and low complication rate.

## Resumo

**Introdução:**

A liquorreia nasal indica uma fístula liquórica, uma comunicação aberta entre o líquido cerebrospinal intracraniano e a cavidade nasal. Pode ser traumática e espontânea.

**Objetivo:**

O objetivo deste estudo foi avaliar o desfecho do reparo endoscópico da fístula liquórica nasal utilizando fluoresceína.

**Método:**

Este estudo retrospectivo incluiu 30 pacientes de ambos os sexos, com idade média de 48,7 anos, tratados de 2007 a 2015. Todos os pacientes foram submetidos à administração lombar de solução de fluoresceína de sódio a 5% no pré-operatório. A fístula foi fechada com enxerto de três camadas e cola de fibrina.

**Resultados:**

As fístulas de líquido cerebrospinal foram comumente localizadas no seio etmoidal (37%) e esfenoidal (33%). A maioria dos pacientes apresentou fístulas liquóricas traumáticas (2/3 dos pacientes). A taxa de sucesso relatada para a primeira tentativa de reparo foi de 97%. Complicações ocorreram em três pacientes: um apresentou hidrocefalia aguda; um, síndrome reversível de encefalopatia no quinto dia de pós-operatório com perda bilateral da visão, e um foi diagnosticado com hidrocefalia dois anos após o reparo de fístula liquórica.

**Conclusão:**

o diagnóstico endoscópico e o reparo de fístulas liquóricas nasais com uso de fluoresceína intratecal tem alta taxa de sucesso e baixo índice de complicações.

## Introduction

Nasal liquorrhea is the leakage of cerebrospinal fluid (CSF) into the nasal cavity. It may be either spontaneous or traumatic. Traumatic CSF leakage usually occurs following basilar skull fractures, or it can appear as iatrogenic after surgical interventions. Spontaneous leakage can be with or without elevated intracranial pressure.^1^, ^2^, ^3^

Although basilar skull fractures are often associated with leakage of the cerebrospinal fluid, Miller initially described spontaneous CSF leakage in a child with hydrocephalus in 1826.^4^ Almost a hundred years later; Cushing^5^ described three cases of surgically treated traumatic CSF leaks. In 1937, Cairns^6^ demonstrated that CSF leaks could also be repaired with extradural application of fascia lata. Until the mid-twentieth century, transnasal approach was reserved for cauterization, when Dohlman^7^ described a transnasal-transethmoidal approach that could seal off leak through the cribriform plate with a septal and middle turbinate flap.

Endoscopic CSF fistula repair is an extracranial extradural approach. It has been accepted worldwide as the method of choice because of excellent visualization, precise graft placement, minimal damage to surrounding tissue, preservation of olfactory function in case of fistula leak through the cribriform plate, shortened operating time and faster recovery time.^8^, ^9^, ^10^, ^11^, ^12^

Intrathecal administration of a 5% fluorescein solution is highly effective in the detection of CSF fistulas. The use of fluorescein gives no false positive results. False negative findings may be due to temporary formation of granulation tissue closing the CSF fistula and preventing the leakage to be visualized at the moment of examination.

The aim of this study was to analyze the success rate of endoscopic repair of cerebrospinal fluid fistulas using intrathecal administration of fluorescein for intraoperative visualization.

## Methods

This retrospective study included 30 patients of both sexes surgically treated for CSF leakage during the period from 2007 to 2015. There were 19 male and 11 female patients with a mean age of 48.7 years. Age ranged between 19 and 68 years. None of the patients had previous CSF fistula repair operation.

**Figure 1:**
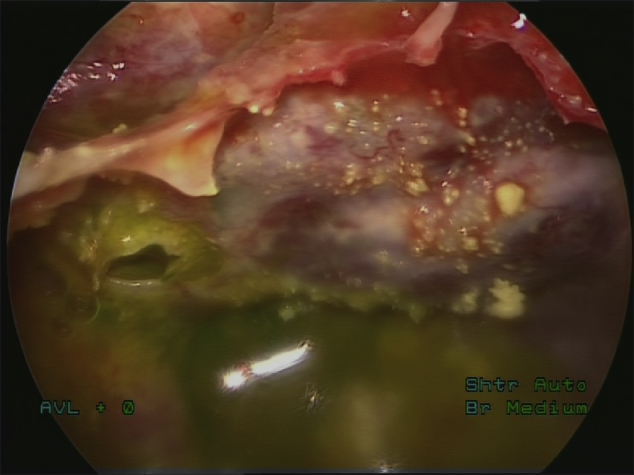
Fluorescein-stained cerebrospinal fluid fistula with a CSF leak, without using a blue light filter.

**Figure 2:**
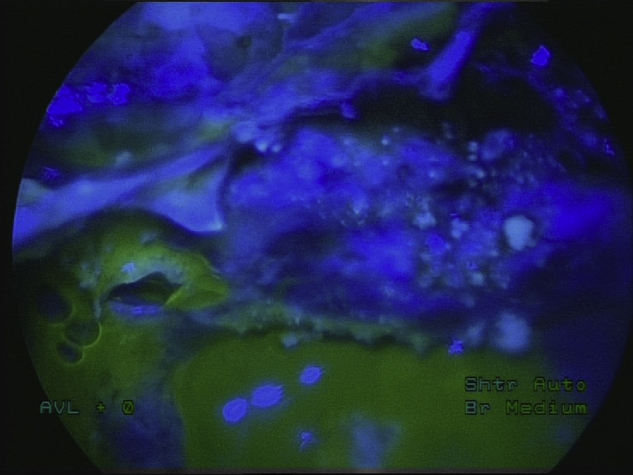
Fluorescein-stained cerebrospinal fluid fistula with a CSF leak, with a blue light filter.

All patients underwent clinical examination, computed tomography (CT) scanning and beta-trace protein test for the diagnosis of nasal liquorrhea preoperatively. Magnetic resonance imaging (MRI) was not routinely performed preoperatively in all examinees; it was indicated in patients with spontaneous CSF leakage, in patients with suspected hydrocephalus and in case of postoperative complications. Lumbar puncture was performed in all patients on the day of surgery by the neurosurgeon. The samples of CSF were taken for biochemical and microbiological analysis. Intrathecal administration of 5% sodium fluorescein solution was done through the performed lumbar puncture one hour prior surgery. Solution of 5% sodium fluorescein was diluted in 5 mL of CSF and gradually injected intrathecally for five minutes with the close monitoring of the patient. During the next one hour prior surgery, patients remained in supine position, lying down on their back and received intravenous antibiotic prophylaxis with 2 g ceftriaxone. The dosage of fluorescein solution was measured according to the body weight (0.1 mL/10 kg of body weight), but the highest dose of intrathecal fluorescein was 1 mL, regardless of the amount of body weight over 100 kg. In order to enhance the visualization of fluorescein, blue light filter (Storz) was used intraoperatively. The appearance of cerebrospinal fluid fistula without using a blue light filter, and with blue light filter is shown in Figure 1, Figure 2.

**Figure 3:**
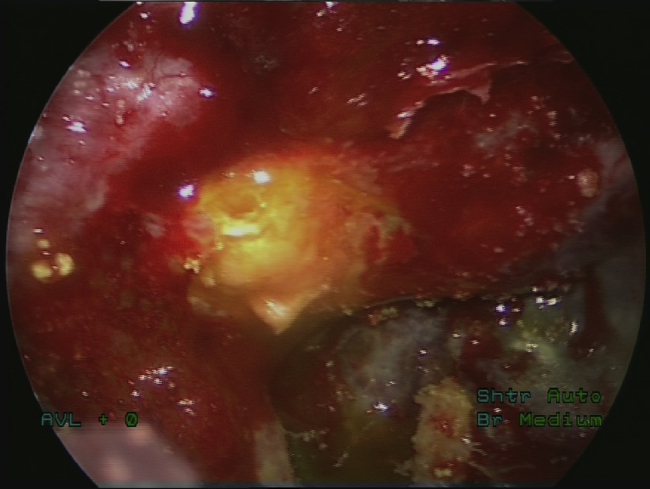
The site of defect after repair of CSF leakage.

Having localized the defect, it was closed using three layers – two layers of fascia lata graft and one mucosal layer (middle turbinate mucosal graft or free nasal mucosal graft) with fibrin glue. The endoscopic repair was performed by the otorhinolaryngologist under general anesthesia. Fig. 3 shows the site of defect after CSF leak management.

After CSF fistula repair, lumbar drain was kept for five postoperative days with complete bed rest. Antibiotics were administered intravenously during this period.

The investigation was done with approval of the Ethics Committee of the institution where the study was carried out (protocol number of Ethics Committee is 00-81/110).

## Results

**Figure 4:**
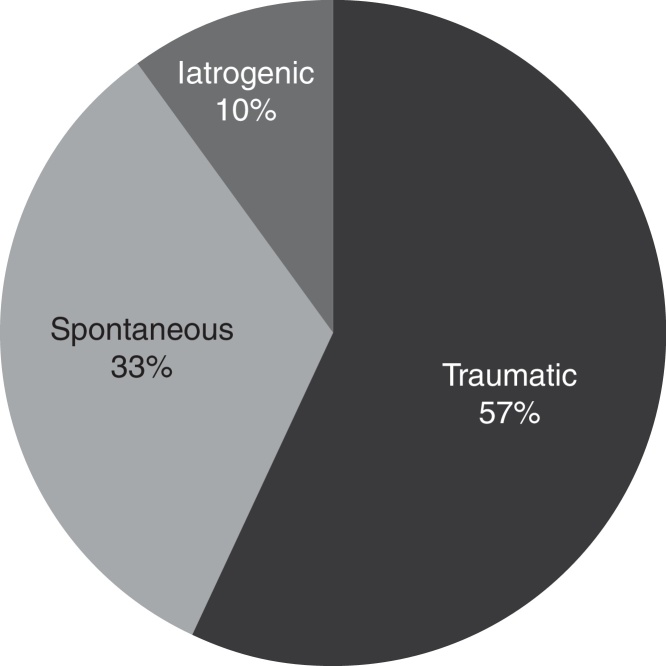
Distribution of etiologic factors of nasal liquorrhea.

Retrospective analysis of the etiologic factors of CSF leaks showed that the largest number of patients had previous head trauma – 17 patients (57%), followed by spontaneous CSF leaks in 10 patients (33%) while 3 (10%) patients previously underwent a surgical procedure (one patient underwent septoplasty, the other one underwent functional endoscopic sinus surgery for chronic rhinosinusitis with polyposis and one patient was operated by the neurosurgeon for olfactory meningioma). Distribution or the patients depending on the etiology of CSF leakage is shown in Fig. 4. In the group of patients with traumatic CSF leakage, most of them were men (14/17); spontaneous CSF leaks occurred predominantly in female patients (7/10) while in the small group of patients with iatrogenic CSF leakage, there were two male and one female patient. Body weight of the examinees ranged between 54 and 116 kg, two patients weighted over 100 kg, with no statistically significant difference in body weight between the groups of patients depending on the CSF leakage etiology.

**Figure 5:**
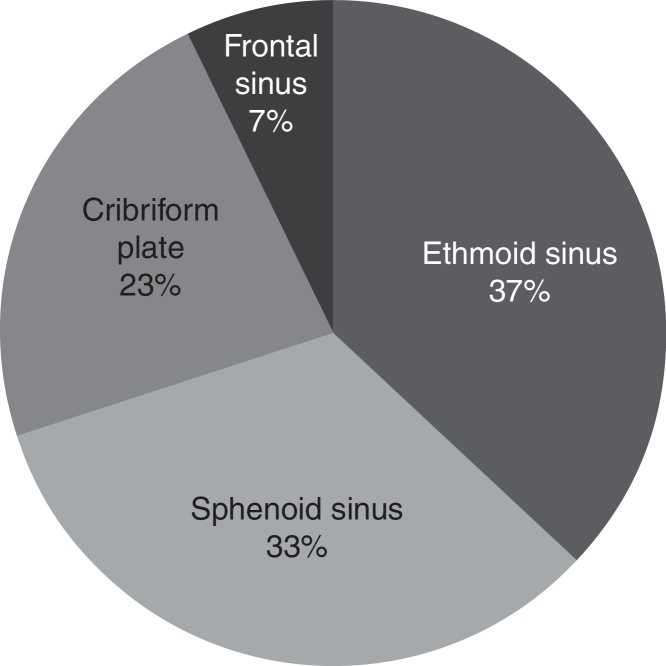
CSF leak fistula locations.

Cerebrospinal fluid leaks were most commonly located in the area of the ethmoid sinus – in 11 patients (37%), followed by sphenoid sinus and cribriform plate as shown in Fig. 5. It is interesting to point out that one patient presented with false nasal liquorrhea. This patient had otoliquorrhea with fistula detected in the basal turn of the cochlea with CSF leakage through the Eustachian tube into the nasal cavity. This patient underwent fistula repair via transmeatal approach and he was not included in this study.

Success rate of endoscopic repair was high (97%) since only one patient had recurrent CSF leakage postoperatively. It developed on his eighth postoperative day. This patient had the CSF leak as a result of previous olfactory meningioma surgery. Due to the development of recurrent CSF leaks and considering that the patient had loss of sense of smell, it was decided, together with neurosurgeons, to perform a transcranial repair of CSF fistula.

In examined group of patients, postoperative complications occurred in three of them; two presented with early, and one with late complications. One male patient, 40 years of age, developed acute postoperative hydrocephalus eight hours after repair of the lateral wall of the sphenoid sinus, and required an urgent neurosurgical intervention (ventriculoperitoneal shunting).

On the fifth postoperative day, one female patient, 49 years of age, developed a Posterior Reversible Encephalopathy Syndrome (PRES), manifesting with bilateral loss of vision. Magnetic resonance imaging showed edema in the occipital region. After conservative antiedema therapy using mannitol and corticosteroids, all symptoms resolved within two days.

Two years after the repair of CSF fistula, one male patient, was diagnosed with hydrocephalus, which was managed with a ventriculoperitoneal shunt.

There were no adverse reactions related to sodium fluorescein administration, whatsoever.

## Discussion

Nasal liquorrhea is a serious condition that may lead to bacterial meningitis by spread of infection from the nasal cavity to the endocranium. During a 10 year period, almost 80% of patients present with at least one attack of purulent meningitis, some even more. Meningitis is more common in patients with traumatic nasal liquorrhea.^12^, ^13^, ^14^

The authors reported no significant difference related to sex distribution, whereas in our sample 2/3 of patients were male, male patients outnumbered female ones in the group of traumatic CSF leaks, while most of the patients with spontaneous CSF leaks were female ones. This may be due to the fact that traffic injuries are more common among males, as well as occupational injuries of male workers whose job includes hard physical labor. Virk et al.^15^ reported 2/3 of the study sample to be women.

More than half of studied patients had a traumatic CSF leakage, and together with those with iatrogenic nasal liquorrhea, it accounts for 2/3 of patients. Kapitanov and associates^16^ reported an equal number of traumatic and spontaneous CSF leaks, while Virk^15^ reported on 2/3 of patients with spontaneous CSF leaks.

The predominance of traumatic CSF leaks in our sample may be due to the fact that our Clinic for ear, nose and throat diseases is a tertiary health institution with close cooperation with the Clinic of Neurosurgery; this is where patients with severe injuries are referred to when they cannot be treated at the secondary level.

Cerebrospinal fluid fistulas are commonly located in the ethmoid (35%), and sphenoid sinus (32%). CSF leaks in the ethmoid sinus may be caused by trauma, either as a part of head trauma or iatrogenic, or they are spontaneous. A highly pneumatized sphenoid sinus may also cause CSF leaks. Extreme pneumatization of the sphenoid sinus may cause bone resorption in the skull base and, sometimes, a prolapse and tearing of the dura. A small meningocele may originate from bone dehiscence in the overpneumatized areas and this pathogenic mechanism should be considered an important etiological factor for spontaneous CSF leak development. Spontaneous CSF leaks are commonly located in the lateral wall of the sphenoid sinus.^17^

Endoscopic endonasal repair of CSF leaks had a high success rate of 97%. This can be attributed to the use of sodium fluorescein solution and blue light filter for detection of cerebrospinal fluid fistula. Only one patient had recurrent CSF leakage which developed on his eighth postoperative day. This patient had the CSF leak as a result of previous olfactory meningioma surgery. Otorhinolaryngologist suggested endoscopic reoperation, but since the patient already had no sense of smell, it was decided, together with neurosurgeons, to perform a transcranial repair of CSF fistula.

Nyquist and associates^18^ studied a similar size sample (28 patients) and reported an overall endonasal closure rate of 93.8% (30 of 32 procedures). Lee and associates^19^ studied a sample of the same size as Nyquist, and reported a success rate of 86% at first-attempt, and 93% at second attempt. Virk et al.^15^ had an overall success rate of 93%, and 100% after the second operation. Lee and associates^19^ believe that the success of endoscopic endonasal repair primarily depends on direct visualization of the defect. Seth and assoc.^20^ emphasize the use of fluorescein: localization of the leak site was greater when fluorescein-colored CSF was visualized, detecting 100% of defects, versus 81.3% without use of fluorescein. Provided that correct fluorescein solution for intrathecal administration is used, in the appropriate dosage, it is not associated with any adverse effects.^21^ Complications were reported when using 10% fluorescein solution for intravenous administration and included vertiginous symptoms, tinnitus, cranial nerve and spinal cord impairment, epileptic seizures.

We believe that use of lumbar drains for 5 days has positive effects on the success of CSF repair, because it reduces the pressure at the point of defect closure; however, some authors do not share this opinion and believe that lumbar drains are necessary only in patients with increased intracranial pressure.^15^ On the other hand, some authors do not use lumbar drains after CSF repair at all.^22^, ^23^ In his study, Oles^24^ did not use lumbar drains, and postoperative management was based on diuretics for 5 days.

Early postoperative complications were present in 2 patients (6%); one male patient presented with acute hydrocephalus eight hours following surgery, which was probably the reason for spontaneously nasal liquorrhea, although preoperative MRI in this patient showed no signs of hydrocephalus and/or altered CSF flow. The second female patient developed a Posterior Reversible Encephalopathy Syndrome (PRES), manifesting with bilateral loss of vision. She was examined by the ophthalmologist, the ocular fundus appeared normal. Endocranial CT was done with no pathology detected, but MRI showed edema in the occipital region. Anti-edematous therapy successfully resolved the symptoms. PRES was described by Hinchey and associates in 1996.^25^ It usually occurs in patients with oscillating blood pressure caused by high blood pressure, renal insufficiency and in those who are immunosupressed. Our patient, unlike these patients, had no comorbidities, her blood pressure was normal. Most of the patients with this syndrome are female, as proven in our study.

One male patient developed late complication, two years following surgery, in the form of hydrocephalus which was managed with ventriculoperitoneal shunt. He also underwent preoperative endocranial MRI showing no abnormalities in CSF flow. MRI is indicated in all patients with non-traumatic CSF leaks in order to detect alteration in CSF flow and in cases of developed postoperative complications.

There were no complications or side effects related to sodium fluorescein.

## Conclusion

Nasal liquorrhea usually occurs in male patients as a result of previous trauma.

Endoscopic detection and repair of cerebrospinal fluid fistulas with three-layer graft and fibrin glue has high success rate and low complication rate.

The use of 5% sodium fluorescein solution for intrathecal administration, in the appropriate dosage, is a safe procedure for detection of cerebrospinal fluid fistulas during endoscopic surgery and causes no adverse effects.

## Conflicts of interest

The authors declare no conflicts of interest.
